# 基于环糊精的农药吸附剂的研究进展

**DOI:** 10.3724/SP.J.1123.2020.08018

**Published:** 2021-02-08

**Authors:** Jinfeng ZHANG, Ping LI, Jiutong MA, Qiong JIA

**Affiliations:** 吉林大学化学学院, 吉林 长春 130012; College of Chemistry, Jilin University, Changchun 130012, China; 吉林大学化学学院, 吉林 长春 130012; College of Chemistry, Jilin University, Changchun 130012, China; 吉林大学化学学院, 吉林 长春 130012; College of Chemistry, Jilin University, Changchun 130012, China; 吉林大学化学学院, 吉林 长春 130012; College of Chemistry, Jilin University, Changchun 130012, China

**Keywords:** 吸附, 环糊精, 农药, 综述, adsorption, cyclodextrin, pesticides, review

## Abstract

农药的研发与使用极大地提高了农作物的产量,为解决人类温饱、改善人类生活品质做出了贡献。但是,农药广泛残留于农副产品以及土壤和水体中,造成的污染日趋严重。残留的农药通常具有微量致毒、难生物降解、生物累积等特性,对生物健康与生态系统造成了巨大威胁。高效检测微量农药、减小污染危害是亟待解决的问题。吸附法具有成本低、操作简单、稳定性强、可重复性强的特点,在农药分离预富集领域得到了广泛关注。作为一种常用的农药吸附剂材料,环糊精是一类具有空腔的超分子化合物,能够作为主体通过主客体作用形成包合物;另外,可以通过醚化、酯化、氧化等化学反应对环糊精进行后修饰以提高其吸附性能。疏水作用、静电作用、范德华力、氢键作用、立体效应协同促进对农药的吸附。环糊精在农药吸附领域已经取得了一定进展,但是目前还没有基于环糊精的农药吸附剂的综述。该文针对杀菌剂、杀虫剂、除草剂、植物生长调节剂这4类农药,系统性地评述了基于环糊精的农药吸附剂的制备、吸附机理及应用,目前存在个别吸附剂吸附容量不高、降解机理不明确、降解产物对环境不友好、容易造成二次污染的问题,研发高吸附容量、易回收、易分离、易再生的基于环糊精的农药吸附剂是未来的主要研究方向。

## Abstract (en)

The invention and application of pesticides have greatly increased the yield of crops, greatly contributing to ensuring people’s basic livelihoods and gradually improving their livelihoods to a well-off level. However, foods, water sources, and soil, containing high levels of pesticide residues, result in increasingly serious pollution. Pesticide residues usually have the characteristics of micro toxicity, difficult biodegradation, and bioaccumulation, and thus pose serious threat to living organisms and ecosystems. In recent years, pesticide pollution has earned worldwide focus. Thus, methods for the efficient detection of trace pesticides and reduction of the harm caused by pesticide pollution are urgently required. Researchers have used catalysis, electrochemistry, membrane separation, adsorption, and other methods to enrich pesticides from complex matrices. Among these, adsorbents have attracted much attention owing to their advantages of simple operation steps, rapid treatment process, and low amounts of organic solvents required. Research on adsorption materials has always been a very active field, and is also the key to the success of separation and enrichment of pesticides from complex matrices. Development of adsorbents with the advantages of simple synthesis, environment-friendliness, high stability, and strong reusability is of great significance. There has been some progress in the field of pesticide adsorption using supramolecular compounds. Cyclodextrin is a macrocyclic compound with a cavity after crown ether, which can form inclusion complexes via host guest interactions as the main body. Cyclodextrin can also be modified by etherification, esterification, oxidation, and other chemical reactions to improve its adsorption performance. Pesticides can be classified into organic and inorganic substances. One of the most widely used inorganic fungicides is the Bordeaux solution, whose main component is Cu^2+^. Organic fungicides, insecticides, herbicides, and plant growth regulators are basically organic molecules, whose hydroxyl and carboxyl groups can form complexes with Cu^2+^. As a matrix, cyclodextrin not only increases the surface area of the materials, but also provides the binding sites of hydroxyl and carboxyl groups, which guarantees efficient enrichment of Cu^2+^. Organic pesticides with high polarity, high electron density, and strong hydrophobicity could be better adsorbed. In this paper, the application of cyclodextrin-based adsorbents in pesticide adsorption was reviewed, and on this basis, reference to future development directions and application prospects were provided. The adsorption capacity of individual pesticide adsorbents based on cyclodextrin, as reviewed in this paper, is not high enough. Therefore, improving the adsorption capacity is currently a major research target. Some of the above-mentioned adsorbents have unclear degradation mechanisms and can easily cause secondary pollution. Therefore, the development of environment-friendly pesticide adsorbents that are easy to regenerate is a promising research direction for the future. After adsorption, some detection methods are used to determine whether the pesticide residues are up to the standard; however, the detection instruments are expensive. Therefore, the development of a combined detection mechanism that can reduce workload and cost is a promising research direction. Finally, the development of smart cyclodextrin-based adsorbents is also an efficient and rapid method to reduce the cost of detecting residual pesticide concentrations and the risk of pesticide pollution. For example, intelligent materials, whose color changes can be observed by the naked eye, not only adsorb pesticides, but also respond according to the concentration of residual pesticides.

农药是指农业上用于防治病虫害及调节植物生长的化学药剂^[1]^。农药的使用极大地增加了作物的产量,改善了作物的品质。目前,已报道的农药种类超过40000种,可以按照原料来源、化学结构、加工剂型、用途等对农药分类。其中,按照用途分类,可以分为杀菌剂、杀虫剂、杀螨剂、杀鼠剂、杀线虫剂、杀软体动物剂、除草剂、植物生长调节剂8类。

随着农药的大量使用,农药残留所引发的问题越来越多。农药广泛残留于农副产品、水体、土壤等,残留的农药通常具有微量致毒、难生物降解、生物累积等特性,对人类的身体健康以及生态环境安全存在直接或潜在的威胁^[2,3,4]^。检出限低、检测时间短、响应速度快、抗干扰能力强的农药残留检测方法一直是研究的热点,色谱法^[5]^、光谱法^[6]^是非常经典的检测方法。但农药残留往往是微量的甚至是痕量的,直接检测效果不佳,在检测前进行分离预富集处理是获得低检出限的有效方法。因此高效的农药富集材料成为研究者们关注的焦点。

近年来,研究者们已利用催化法^[7]^、电化学法^[8]^、膜分离法^[9]^、吸附法^[10]^等方法用来分离富集农药。其中吸附法操作步骤简单,处理过程快速,有机溶剂用量少,且可以富集复杂样品中存在的痕量农药,以达到快速检测、高效去除样品中残留农药的目的,是目前农药富集领域广泛使用的技术^[11,12,13]^。在吸附法中,吸附材料的研究一直是十分活跃的领域,既是吸附分离成败的关键,也是检测痕量农药残留的关键,开发合成过程简单、环保、稳定性高、可重复性高、成本低的吸附剂对于农药的高效检测和吸附具有重要的意义。

超分子化合物在吸附领域已经取得了一定进展^[14,15,16,17]^。环糊精(CD)是超分子化合物的一个分支,是继冠醚之后出现的一类具有空腔的大环化合物。环糊精能够作为主体通过主客体作用形成包合物;另外,还可以通过醚化、酯化、氧化等化学反应对环糊精进行后修饰,以提高其吸附性能^[18,19,20]^。近年来,环糊精在分离预富集领域得到了极高的关注,被广泛报道用作农药的吸附剂。本文基于环糊精吸附剂在农药吸附领域的应用进行综述,并在此基础上对今后的研究加以展望,为其在农药分离富集研究领域的应用提供参考和思路。

## 1 环糊精概述

环糊精是直链淀粉在由芽孢杆菌产生的环糊精葡萄糖基转移酶作用下生成的一系列环状低聚糖的总称,通常含有6~12个D-吡喃葡萄糖单元。其中研究的较多并且具有重要实际意义的是含有6、7、8个葡萄糖单元的分子,分别为*α*、*β*、*γ*-环糊精(*α*、*β*、*γ*-CD)^[21]^。环糊精的结构(见图1)是呈锥形的圆环,其空腔受到碳氢键的屏蔽作用而具有疏水性,且能通过主客体作用将客体分子捕捉到空腔内。大开口端与小开口端上的羟基使环糊精具有一定的亲水性和还原性,也使得其容易被改性。环糊精的独特性质使其自被发现以来就成为研究者们关注的热点。

**图1:**
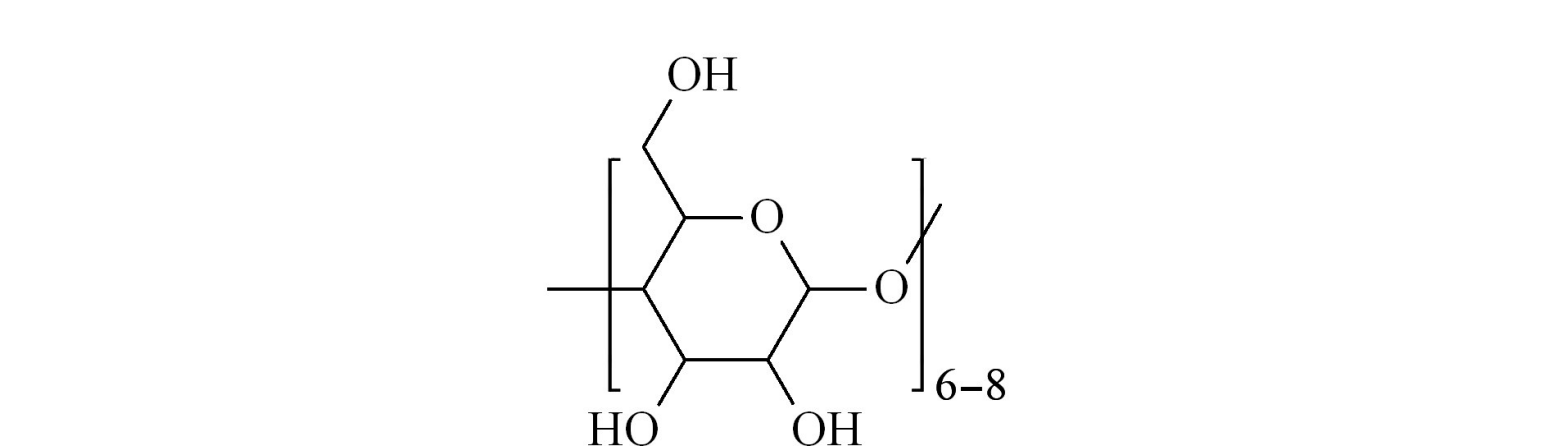
环糊精的结构

环糊精由于其独特的化学结构和化学性质,被广泛应用于分离富集领域。环糊精及其衍生物在分离手性物质^[22,23,24,25]^、生物医学^[26,27]^、疾病诊疗^[28]^,以及选择性吸附气体^[29,30,31,32,33]^、无机金属离子^[34,35,36,37,38,39,40]^、药物^[41]^、糖蛋白/肽^[42,43,44,45,46]^、环境污染物^[37,47-53]^等方面已经取得了一定进展。例如,Wang课题组^[54]^首次将甲基丙烯酸改性的*β*-CD与1-乙烯基咪唑(VI)在不同单体投料比下共聚,制备了一系列*β*-CD交联吸附剂(PCD-VI),有效避免了有机染料与重金属离子的竞争吸附。PCD-VI对罗丹明B、刚果红、Cd^2+^的最大吸附量分别为336、1062和117 mg/g,且5次循环后吸附性能不会明显降低。该吸附剂抗干扰能力强,Na^+^、K^+^、Mg^2+^、Ca^2+^对吸附过程无干扰。Jia课题组^[42]^制备了花生凝集素(PNA)改性的*β*-CD化合物(PNA-*β*-CD),利用化学键合手段将PNA-*β*-CD修饰到聚(甲基丙烯酸羟乙酯-co-乙二醇二甲基丙烯酸酯)整体柱上,随后将其用于聚合物整体柱微萃取装置,并与质谱仪进样口连接,进行在线解吸检测复杂样品中的半乳糖糖基化糖肽。在最佳实验条件下,分别应用于检测血液样品和人急性髓系白血病细胞(Molm-13)样品中的半乳糖糖基化肽段。该方法的检出限为0.5 fmol,整体柱批次与批间精密度分别为1.3%和4.2%,基质抗干扰效应为(0.85~1.21)±0.03。将富集后得到的人血清样品糖肽数据通过数据库搜索软件解析,鉴定到137个*N*-糖基化位点和对应的101种糖蛋白。该课题组^[41]^随后以*β*-CD为反应单体,以4,4'-二氯甲基-1,1'-联苯作为交联剂,制备了一种基于环糊精的超交联聚合物。该材料具有较高的比表面积和良好的热稳定性,对阿苯达唑的吸附过程符合伪二级动力学模型和Langmuir等温线模型,最大吸附量达到了181.82 mg/g。此外,在多次循环吸附后,该材料的吸附能力没有明显降低。环糊精与客体分子之间具有主客体作用,因此基于环糊精的吸附富集材料实现了高效、特异性的分离富集。在农药的分离预富集方面,环糊精基吸附材料也取得了非常可观的成果,下文就环糊精在农药吸附领域的应用进行了综述。

## 2 环糊精在农药吸附领域的应用

杀菌剂、杀虫剂、除草剂、植物生长调节剂是使用量较大的农药,杀鼠剂、杀线虫剂、杀软体动物剂使用量较小,而且杀菌剂、杀虫剂也在一定程度上起到了杀鼠、杀线虫、杀软体动物的作用。因此,本文从杀菌剂、杀虫剂、除草剂、植物生长调节剂这个方面对基于环糊精的农药吸附剂进行总结阐述。

### 2.1 杀菌剂

真菌、强菌、立克次氏体、支原体、病毒、藻类等对农业造成了巨大的损失,历史上一些植物病毒的流行轻则使农作物减产,重则造成严重饥荒^[55]^。施用杀菌剂对植物进行杀菌是一种经济高效的方法。杀菌剂可以分为无机杀菌剂和有机杀菌剂两类。

2.1.1 无机杀菌剂

波尔多液是目前施用量最大的一种无机杀菌剂,1878年首次被发明,至今仍被广泛使用。波尔多液由硫酸铜、生石灰、水按照一定比例配制而成,其中铜离子(Cu^2+^)使蛋白质变性以达到杀菌的目的,但同时也会对水体、土壤造成污染。因此,对波尔多液的去除主要是吸附Cu^2+^。

羟基、羧基可以与Cu^2+^形成络合物,环糊精既可以增加材料的表面积又可以提供羟基、羧基的结合位点,极大地丰富了吸附剂表面的羟基、羧基。一些课题组基于这个原理,利用接枝、共沉淀等方法对环糊精进行后修饰,合成了一系列环糊精聚合物。如Wang等^[56]^通过酰化反应合成环糊精丙烯酰氯(*β*-CD-Ac),将甲基丙烯酸缩水甘油酯(GMA)接枝到二氧化硅(SiO_2_)与*β*-CD-Ac表面,合成的水凝胶(SiO_2_-g-GMA/*β*-CD-Ac)是一种高效、专一的Cu^2+^吸附剂,吸附容量为110.0 mg/g。该水凝胶呈球形,大的比表面积及表面上键合的多羟基提供了Cu^2+^结合位点。该吸附过程是放热的,适当的低温能达到更大的吸附容量。

Li等^[57]^通过酯化反应将环糊精和柠檬酸接枝到普通滤纸上,合成了改性滤纸(MFP)。普通滤纸由于含有大量纤维素,接枝反应较容易发生。MFP合成过程相对简单,机械强度远高于普通滤纸,且结合了吸附与过滤的优点。吸附动力学符合拟二级模型,主要受化学吸附控制,Cu^2+^的吸附容量为58.79 mg/g, 20 mim去除率即达到80%,且30 min左右达到吸附平衡,吸附速率快。Kameda等^[58]^采用共沉淀法制备了羧甲基改性环糊精(CMCD)离子插层的Zn-Al层状氢氧化物(CMCD-Zn-Al-LDHs),吸附容量为53.98 mg/g。动力学与热力学表明,CMCD-Zn-Al-LDHs吸附Cu^2+^的反应在传质控制下进行,CMCD-Zn-Al-LDHs层间的大量羟基与Cu^2+^之间形成了非常稳定的螯合物,吸附效率较高。

吸附剂与四氧化三铁(Fe_3_O_4_)磁性粒子结合,可以通过磁铁快速、方便地实现分离过程^[59]^。Badruddoza等^[60]^采用碳二亚胺法将CMCD接枝到Fe_3_O_4_表面,合成了一种吸附Cu^2+^的高效吸附剂(CMCD-MNPs)。Cu^2+^与CMCD-MNPs表面的氧原子形成络合物,接枝的多羟基、多羧基是高效吸附的保证。该吸附剂吸附动力学符合准二级动力学模型,热力学符合Langmuir等温模型。在25 ℃时,Cu^2+^的最大吸附量是47 mg/g,吸附速率快,30 min即可达到吸附平衡;吸附过程与温度、溶液的pH值有关,理论上通过调节温度和溶液的pH值能获得更好的吸附容量。柠檬酸作为洗脱剂,解吸率为96.2%,由钕铁硼制成的永磁铁可以快速地回收CMCD-MNPs,且CMCD-MNPs与洗脱剂的上清液分离后,上清液中的Cu^2+^可以二次利用。

Ansari等^[61]^合成了磁性羟基磷灰石-环糊精吸附剂(Fe_3_O_4_@HA-*β*-CD),羟基磷灰石接枝在环糊精表面,增大了吸附剂的表面积,丰富了羟基的含量,吸附动力学数据符合拟二级模型,吸附等温线符合Langmuir模型。Fe_3_O_4_@HA-*β*-CD与Cu^2+^接触1 h后达到吸附平衡,对Cu^2+^的最大吸附量为66.66 mg/g,经过5次循环之后,吸附剂的吸附容量只下降了19%。乙二胺四乙酸二钠(EDTA)作为该材料的洗脱剂,Cu^2+^释放率为88%。外加磁场即可回收Fe_3_O_4_@HA-*β*-CD,无二次污染。

含有多个环糊精单元衍生物的环糊精聚合物也是性能优良的吸附剂^[41,51]^。Huang等^[62]^合成了柠檬酸交联环糊精聚合物吸附剂(CA-*β*-CD),表面交联的羧基提供了Cu^2+^的结合位点,非均相体系中Cu^2+^的吸附容量是58.13 mg/g。腐殖酸是天然有机物的主要成分,是实际应用中最大的干扰因素,实验中发现腐殖酸对该材料的吸附性能无影响,且该材料在1 min内就能完成吸附并沉降。CA-*β*-CD合成过程简单,对环境友好,并且具有极好的可回收性和抗干扰能力,有很大的实际应用潜能。Chen等^[63]^以环糊精为基料,制备了一种环保型聚多巴胺复合吸附剂(CD-CA/PDA)。CD-CA/PDA结合了环糊精和聚多巴胺的优点,具有相当丰富的羧基和邻苯二酚基团,因此Cu^2+^可以很容易地被吸附。吸附动力学结果表明,吸附过程动力学符合拟二级模型。此外,等温线拟合结果表明,CD-CA/PDA在Cu^2+^上的吸附过程符合Sips模型。CD-CA/PDA使用条件简单,在pH 4~12之间都可以完成吸附,具有良好的可回收性,至少可重复使用5次。

巯基与Cu^2+^也可以形成络合物,利用巯基修饰环糊精也是一种新的研究思路。通过一定手段修饰环糊精可以作为硫离子(S^2-^)的缓释剂,使Cu^2+^均匀沉淀。该沉淀溶解度极小,过滤即可除去。其他超分子化合物如冠醚、杯芳烃、葫芦脲、柱芳烃等和环糊精性质类似,可以作为载体并通过化学反应进行改性,也为降低波尔多液的危害提供了新的方法。

上述吸附剂完成吸附后,可通过原子吸收光谱法检测处理后的土壤、水样、农副产品中农药残留量是否达标,原子吸收光谱法是检测Cu^2+^的常用方法^[57]^。

2.1.2 有机杀菌剂

有机杀菌剂种类较多,研究者们已利用催化法^[64,65,66,67,68]^降解有机杀菌剂,且该方法已接近成熟,而吸附法的应用较少。本文以嘧菌酯、百菌清、肟菌酯、嘧啶环胺、三唑酮为例进行阐述。其中,嘧菌酯、百菌清、肟菌酯是高效、广谱的杀菌剂,对所有的真菌都有一定的杀菌作用。嘧啶环胺是植物性杀菌剂,对种子有保护作用。三唑酮俗名为粉锈宁,对由真菌引起的锈病、白粉病有很好的治疗效果。

Yang等^[69]^合成了交联1-己基-3-甲基咪唑双三氟甲磺酰亚胺盐的磁性环糊精硅镁土吸附剂([HMIM]NTF_2_-M-*β*-CD/ATP)。该吸附剂对嘧菌酯、百菌清、嘧啶环胺、肟菌酯的富集倍数分别是143、135、151和159倍,且2.5 min即可完成吸附。原料成本较低,易于被回收,于丙酮中清洗两次并置于50 ℃下的真空烘箱中,8 h即可再生。利用该吸附剂对池水、溪水、雨水和河水中的嘧菌酯、百菌清、嘧啶环胺、肟菌酯进行富集,通过高效液相色谱仪进行分析,检出限为0.02~0.04 μg/L,回收率为81%~109%。证明该吸附剂具有很大的实际应用潜能。

Senosy等^[70]^采用环糊精对金属有机骨架进行后修饰,四氟对苯二甲酸作为交联剂,合成了磁性吸附剂(Fe_3_O_4_@MIL-100(Fe)/*β*-CD)。由于组装体中环糊精的独特结构,Fe_3_O_4_@MIL-100(Fe)/*β*-CD复合材料能与目标农药形成主客体包合物,极大地提高了吸附性能;Fe_3_O_4_@MIL-100(Fe)/*β*-CD的磁性有助于快速、方便地进行磁选,提高分离效率;Senosy等^[70]^使用Welch Ultimate^®^ XB-C18柱(250 mm×4.6 mm, 5 μm)在30 ℃下通过高效液相色谱实现分离。实验条件:流动相为甲醇-水(70∶30, v/v),流速为1.0 mL/min,进样体积为20 μL,检测波长为230 nm。通过数据分析结果可知,废水样品中三唑酮、多效唑、氟硅唑、4-戊唑醇的去除效率为74.90%~100.00%,湖泊水样中三唑酮、多效唑、氟硅唑、4-戊唑醇的去除效率为86.14%~99.70%, 50 min即可完成吸附,5次循环后去除效率基本不变。

在上述报道中,吸附原理都是环糊精与有机杀菌剂形成主客体包合物。环糊精的空腔与有机杀菌剂的匹配程度影响吸附效率。若是农药分子较小,可以选择空腔相对较小的*α*-CD作为基料,若是农药分子较大,可以选择空腔相对较大的*γ*-CD作为基料。

### 2.2 杀虫剂

蝼蛄、蛴螨、菜青虫、叶螨、蚜虫、蜱虫等上百种昆虫严重影响农作物的生长,甚至会造成农作物的死亡。为了降低农业害虫的危害,研究者们发明了有机氯、有机磷、氨基甲酸酯类、新烟碱类、苯甲酰脲类等杀虫剂。杀虫剂对害虫有较好的防治效果,但是在保护农作物的同时对生物的健康威胁也是不可忽视的。

2.2.1 有机氯杀虫剂

氯元素的强氧化性能够杀死害虫,且有机氯农药性质稳定,杀虫效果非常好。毒性强、不易降解是有机氯杀虫剂的特点^[71,72]^。为了解决上述问题,Salazar等^[73]^合成了纳米Fe_3_O_4_修饰的环糊精聚合物,以去除水中芳香族氯代农药。采用紫外可见光谱法对完成吸附后的水样进行检测,4-氯苯氧乙酸、2,3,4,6-四氯苯酚的浓度为0.01 mmol/L,吸附剂与污水样接触时间为360 min时,4-氯苯氧乙酸的吸附容量为0.59 mg/g,可以去除91%的4-氯苯氧乙酸;2,3,4,6-四氯苯酚的吸附容量为0.63 mmol/g,可以去除78%的2,3,4,6-四氯苯酚。该吸附剂循环8次,吸附容量基本保持不变。

2.2.2 有机磷杀虫剂

有机磷杀虫剂是广谱性的杀虫剂,遇水易降解,残留小,杀虫剂与其降解物对神经系统有很大毒性^[74,75]^。Schafer等^[76]^用聚醚砜(PES)和环糊精合成了聚合物复合电纺丝纳米纤维(见图2)。以5 mg/L毒死蜱为模型污染物,测定其吸附潜力。复合电纺丝纳米纤维比裸露纤维在恒定重量的情况下纤维的长度更长,表面积更大。环糊精与杀虫剂形成包合物增强了复合电纺丝纳米纤维对毒死蜱的吸附能力。与裸PES纳米纤维相比,毒死蜱吸附量增加了80%。用水冲洗完成吸附的复合电纺纳米纤维后,水中毒死蜱的浓度没有明显升高,对水无二次污染,经过4次循环吸附量基本不变。乙醇作为解吸剂,解吸完成后即再利用。Moon等^[77]^将有机磷水解酶作为聚环糊精的涂层,合成了一种新型的既能吸附甲基对氧磷(MPO)又能自净化的材料。环糊精通过疏水作用与MPO形成主客体包合物,又是有机磷水解酶涂层的载体;二者协同促进了MPO的降解。20 min即可达到吸附平衡,吸附容量为1.26 mg/g,连续使用4 d吸附容量不变,在中和有机磷农药和环境保护领域有很大的潜能。

**图2:**
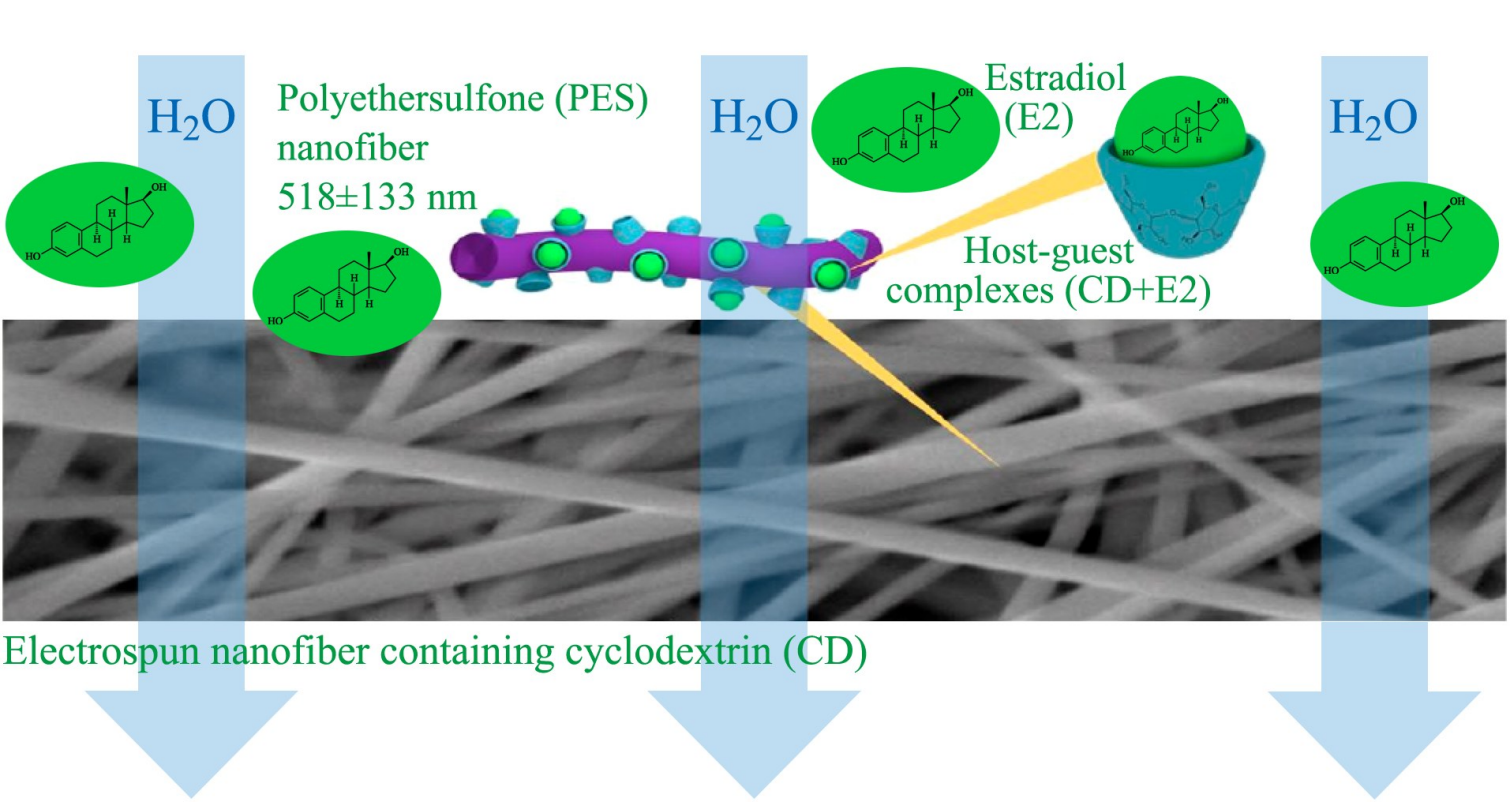
环糊精聚合物复合电纺丝纳米纤维^[76]^

2.2.3 氨基甲酸酯类杀虫剂

氨基甲酸酯类杀虫剂是氨基甲酸的衍生物,性质稳定,能长时间发挥杀虫作用,其中用量较多的是抗蚜威、西维因、呋喃丹。氨基甲酸酯类杀虫剂作用机理与有机磷农药类似,都是胆酯酶抑制剂,对生物体的神经系统有很大危害^[78]^。一些课题组已经合成了特异性富集西维因、呋喃丹、抗蚜威的吸附剂。He等^[79]^利用二元功能单体甲基丙烯酸(MAA)或丙烯腈(AN)制备了可以选择性识别并大量富集抗蚜威的基于*β*-环糊精的分子印迹聚合物(MIPs)。当水和甲醇的比例为95∶5(v/v)时,MAA的吸附容量从13.05 mg/g增加到16.55 mg/g, AN的吸附容量从10.33 mg/g增加到14.73 mg/g。另外,通过增加水的体积以降低混合溶剂中甲醇的含量,可以在溶剂中产生更强的疏水性,从而使抗蚜威很容易进入*β*-CD的空腔,形成主客体包合物。MIPs的立体形状效应和疏水相互作用决定了对抗蚜威的高吸附容量。Ma等^[80]^利用多面体倍半硅氧烷(POSS)和Fe_3_O_4_对环糊精进行修饰,合成了可以吸附西维因、呋喃丹的纳米材料(*β*-CD@POSS@Fe_3_O_4_),建立了磁固相萃取-高效液相色谱法测定苹果中西维因和呋喃丹的方法。该方法对西维因和呋喃丹在2~400 μg/kg范围内具有良好的线性关系,相关系数为0.9995。苹果中西维因和呋喃丹的检出限分别为0.5 μg/kg和0.7 μg/kg。萃取回收率为94.2%~103.1%,可用于痕量西维因和呋喃丹的测定。随着分析物含量(5.0~60.0 μg/mL)的逐渐增加,吸附容量也逐渐增大,25 min即可达到最大吸附量,对西维因和呋喃丹的吸附量分别为27.22 mg/g和19.53 mg/g。该纳米粒子具有良好的长期稳定性,微粒重复使用5次,西维因和呋喃丹的回收率没有显著下降;放置1、3、6、12个月后,吸附回收率没有显著降低。含0.01%磷酸的甲醇和乙腈可以达到相同的洗脱效果,洗脱剂用量少且洗脱时间短,考虑到毒性与成本,以甲醇为洗脱剂,1.0 mL洗脱液20 min即可完全洗脱,回收率没有明显降低。*β*-CD中的羟基与西维因和呋喃丹分子中的氮或氧原子形成氢键,*β*-CD的疏水空腔包合西维因和呋喃丹中的萘或苯环,同时POSS的立方刚性结构提供了疏水环境,西维因和呋喃丹可通过范德华、氢键、疏水作用保留在吸附剂表面。西维因的疏水性比呋喃丹大得多,所以相对于呋喃丹,*β*-CD@POSS@Fe_3_O_4_对西维因的吸附容量更大。这表明该材料对疏水性更强的农药可能有更好的吸附效果。

2.2.4 新烟碱类杀虫剂

新烟碱类杀虫剂是人工合成的烟碱衍生物,对害虫的灭杀作用特别强。一些课题组已研发了相应的吸附剂以降低残留的新烟碱类杀虫剂的危害。Liu等^[81]^将铜基金属有机骨架作为Fe_3_O_4_氧化石墨烯-环糊精的涂层,用于吸附去除水中的新烟碱类杀虫剂,利用高效液相色谱-串联质谱法判断吸附剂的富集能力。该材料表面积为250.33 m^2^/g,吸附能力强,噻虫嗪、吡虫啉、啶虫脒、烯啶虫胺、二口恶英、噻虫胺、噻虫啉的吸附容量分别为100、2.88、3.11、2.96、2.56、1.77和2.88 mg/g。噻虫嗪的吸附等温线符合Langmuir模型,其他6种的吸附等温线符合Freundlich模型,上述7种杀虫剂都含有疏水基团、离域大*π*键、苯环、五元杂环、疏水基团以及高的电子密度,这些特点都增强了该吸附剂与杀虫剂的吸附作用。Salazar等^[82]^合成了超顺磁性Fe_3_O_4_纳米粒子修饰的环糊精基碳酸盐纳米复合材料,以除去新烟碱类杀虫剂二口恶英;环糊精捕获二口恶英到疏水空腔内,120 min即可达到最大吸附量1.46 mg/g,通过紫外可见光谱检测数据可知去除效率可达90.3%,经过8次吸附和解吸循环后,吸附容量仍保持不变,通过外加磁场即可回收。

2.2.5 苯甲酰脲类杀虫剂

苯甲酰基脲类化合物是苯甲酰脲类杀虫剂的主要成分,通过抑制几丁质合成,导致昆虫死亡,对鱼类等生物有一定毒性^[83]^。Yang等^[84]^通过直接浸入法将1-辛基-3-甲基咪唑双三氟甲基磺酰亚胺([HMIM]TF_2_)固定在环糊精基硅镁土(ATP)材料上,合成了高效富集氟铃脲、氟芬隆、氯芬奴隆、氟脲4种农药的吸附剂。氟铃脲、氟芬隆、氯芬奴隆、氟脲的富集倍数分别是121、112、147和150倍。ATP接枝在*β*-CD表面,增强了*β*-CD与上述4种杀虫剂的疏水效应、氢键作用、*π-π*作用,[HMIM]TF_2_提供了更强的疏水环境,二者协同增加了该吸附剂的吸附容量,此吸附剂极容易再生,2 min内用乙腈即可完成农药洗脱和吸附剂再利用。在最佳条件下,检出限为0.12~0.21 μg/L。在5~500 ng/mL范围内,相关系数为0.9997~1.0000,加标回收率为84.5%~104.7%。

考虑到用于去除杀虫剂的环糊精吸附剂的作用机理,极性较大、电子密度较大、疏水性较强的农药能被更好地吸附。其次,环糊精本身具有疏水空腔,通过后修饰将疏水基团键合在*β*-环糊精上可以提高吸附剂的性能。

### 2.3 除草剂

除草剂按照化学成分可分为吡啶类、酰胺类、咪唑啉酮类、有机磷类、醚类、取代脲类、苯氧乙酸类、酚类等。其中,吡啶类、酰胺类、咪唑啉酮类、有机磷类除草剂除草效果好,但造成的污染也较多。有机磷类除草剂中最常用的是草甘膦,降解草甘膦最常用的方法是催化法,如光催化^[85,86]^、酶催化^[87]^、生物催化^[88]^等。本文将对吡啶类、酰胺类、咪唑啉酮类除草剂的*β*-环糊精吸附法进行具体阐述。

2.3.1 吡啶类除草剂

百草枯是吡啶类除草剂的一种,几乎可以除去所有的杂草。但是百草枯毒性极强,对生物体健康和环境危害极大。研究者们探寻了多种基于环糊精的百草枯吸附剂,以降低残留百草枯的危害。

百草枯分子可以进入环糊精的空腔并且被三维交联聚合物的网络捕获,另外,由于百草枯属于阳离子农药,接枝在环糊精表面的阴离子通过静电作用增强对百草枯的吸附能力。基于上述原理,Junthip^[89]^通过环糊精与1,2,3,4-丁烷三羧酸的交联反应合成了阴离子环糊精聚合物,以吸附百草枯。利用紫外分光光度法(检测波长为257 nm)检测百草枯的含量,在pH值为8、温度为30 ℃的最佳条件下,当百草枯初始浓度增加(10、50和250 mg/L)时,吸附容量随之增加(5.0、20.4和25.9 mg/g), 420 min达到吸附平衡。吸附动力学符合拟二级模型,吸附等温线符合Langmuir模型。经过4次循环使用,改性织物在甲醇中的回收率达到85%。甲醇作为洗脱剂降低了该材料的成本,为该改性织物的广泛应用打下了基础。随后,该课题组^[90]^又将阴离子环糊精聚合物与柠檬酸在180 ℃下交联30 min,以消除水中的百草枯。在最佳的吸附条件下,120 min内即可达到吸附平衡。在30 ℃时,当百草枯初始浓度不断增加(25、50、200 mg/L)时,吸附容量也不断增大(9.4、17.4、20.8 mg/L)。吸附动力学符合拟二级模型,吸附等温线与Langmuir模型吻合。在甲醇溶液浸泡120 min即可解吸,4次再生循环后,不溶性聚合物的重复使用率达到78.3%。

2.3.2 酰胺类除草剂

酰胺类除草剂由于毒性相对低,且除草效果好,是目前应用最广泛的除草剂。酰胺类除草剂电子密度较大,环糊精的*π-π*相互作用、氢键作用和静电作用促进了其对农药的吸附,吸附剂的高表面积与多孔性也可以提高吸附容量。Alsbaiee等^[91]^通过环糊精与刚性芳香基的交联反应,合成了一种高表面积的介孔聚合物(P-CDP)。高比表面积和永久孔隙率是快速吸附和高吸附容量的基础,其三维空腔能特异性地包合非平面化合物,吸附容量为26 mg/g,同时可以快速去除部分极性有机污染物。通过高效液相色谱-质谱法定量分析异丙甲草胺在μg/L浓度下的去除率,6 min即可除去92%的异丙甲草胺。另外,洗脱条件温和,P-CDP可以再生且性能不会明显降低。Liu等^[92]^合成了环糊精-金属有机框架衍生的多孔碳材料(*β*-CD-MOF-NPC),该材料具有微孔结构,比表面积大,热稳定性强,通过*π-π*相互作用、氢键和静电作用诱导吸附除草剂(见图3)。异丙甲草胺、甲草胺、乙草胺、丙草胺的吸附容量分别是343.42、291.26、291.26和311.78 mg/g。Liu等^[92]^通过高效液相色谱法和超高效液相色谱-串联质谱法测定农药残留量以评估*β*-CD-MOF-NPC的富集能力。即使上述4种除草剂含量低至10 μg/L或100 μg/L, 5 d内也可以完全除去。

**图3:**
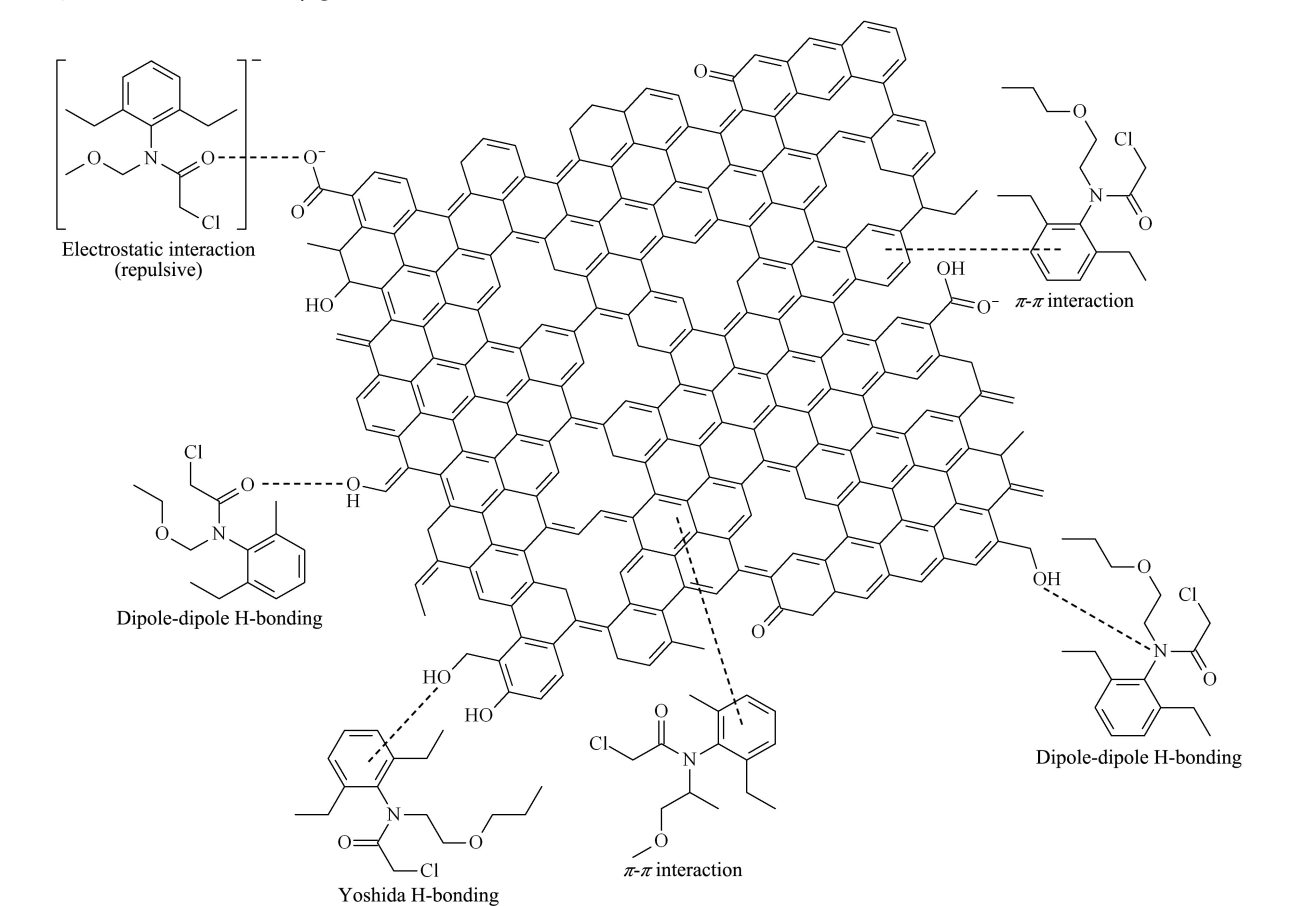
*β*-CD-MOF-NPC与4种酰胺类农药的作用机理^[92]^

2.3.3 咪唑啉酮类除草剂

咪唑啉酮类除草剂是广谱、高效的除草剂,主要应用于大豆除草。Paawan等^[93]^合成了壳聚糖-环糊精生物复合材料,旨在净化土壤中残留的咪唑乙烟酸和甲氧咪草烟。壳聚糖加快了该吸附剂进入土壤溶液的速度,环糊精将咪唑乙烟酸、甲氧咪草烟捕获进空腔,形成包合物。采用液相色谱-串联质谱法对咪唑乙烟酸、甲氧咪草烟进行定量分析。最佳条件下,在0.01、0.1、1、10 μg/mL的初始除草剂浓度下,对咪唑乙烟酸、甲氧咪草烟去除率分别为63.90%~99.44%、62.96%~99.35%、61.36%~99.08%和59.42%~98.59%。但是该材料完成吸附后的降解机理不是很明确,可能存在二次污染。因此,在设计吸附农药的吸附剂时,不仅要考虑农药的危害,也要考虑吸附剂本身及其降解物的污染。

### 2.4 植物生长调节剂

植物生长调节剂是一类用于促进生根、发芽、生长、成熟,调节植物生长发育的农药。植物生长调节剂低毒高效,经过一段时间,残留在土壤、水源中微量的植物生长调节剂即可降解,对土壤、水源的污染较小。但很多不法商家为了追求商业价值滥用植物生长调节剂,农副产品中高残留的植物生长调节剂对人体代谢具有潜在威胁。

基于Fe_3_O_4_超顺磁性与氧化石墨烯(GO)大表面积的特点,键合Fe_3_O_4_、GO的环糊精农药吸附剂的研发已引起研究者的兴趣。如Chen等^[94]^合成了环糊精改性的磁性氧化石墨烯复合材料(Fe_3_O_4_@SiO_2_/GO/*β*-CD),该材料与氧化石墨烯复合材料(Fe_3_O_4_@SiO_2_/GO)相比,可特异性吸附阿特拉津、三唑酮、多效唑、戊唑醇。当阿特拉津、三唑酮、多效唑、戊唑醇含量分别为20、10、10和10 μg/kg时,Fe_3_O_4_@SiO_2_/GO/*β*-CD与Fe_3_O_4_@SiO_2_/GO对上述4种农药的吸附能力的比值分别为1.42、1.50、1.61和2.38。阿特拉津、三唑酮、多效唑、戊唑醇都含有芳香环,*β*-CD的空腔能与芳香环形成特异性的包合物,这决定了Fe_3_O_4_@SiO_2_/GO/*β*-CD对上述4种物质的吸附性能优于Fe_3_O_4_@SiO_2_/GO。该吸附剂与气相色谱-质谱仪联用对蔬菜中残留的植物生长调节剂进行富集检测,在1~100 μg/kg范围内,相关系数为0.9983~0.9996,检出限为0.04~0.28 μg/kg。

利用Fe_3_O_4_表面包覆SiO_2_层,来提高铁磁抗氧化能力及阻止Fe_3_O_4_聚集,再利用GO增加吸附剂的表面积。Cao等^[95]^合成了一种高效的可选择性富集植物生长调节剂的吸附剂(Fe_3_O_4_@SiO_2_/GO/*β*-CD/IL),对2-萘氧乙酸、2,3,5-三碘苯甲酸、*N*6-(2-异戊烯基)-腺嘌呤、6-苄基氨基嘌呤、吲哚菲、烯效唑、戊唑醇7种植物生长调节剂具有很高的吸附容量。Fe_3_O_4_@SiO_2_/GO/*β*-CD/IL的表面积和孔隙总体积分别为55.44 m^2^/g和0.22 cm^3^/g,高的表面积和大孔隙的特性保证了良好的吸附容量。环糊精的空腔可以特异性的捕获含有芳香环的物质,离子液体(IL)中阴离子的芳香结构可以进一步增强吸附剂与上述7种植物生长调节剂之间的*π-π*相互作用,促进了吸附容量的提高。该材料吸附速率快,5 min即可完成吸附,吸附剂的超顺磁性有利于回收。与高效液相色谱-三重四极杆离子阱质谱联用,Cao等^[95]^测定了蔬菜样品中7种植物生长调节剂,在2~50 μg/kg范围内,线性关系良好,相关系数大于0.9982,检出限为0.01~0.18 μg/kg。

## 3 总结与展望

环糊精对农药的吸附主要通过空腔的主客体作用与农药形成包合物,再利用疏水作用、静电作用、范德华力、氢键作用以及立体效应的协同作用提高吸附富集效果,为痕量农药的检测工作提供了基础。基于环糊精的吸附剂吸附性能好,可重复利用性强,成本低,无二次污染,在农药分离预富集领域具有很好的应用前景。但是仍存在一些弊端,如前文综述的个别吸附剂样品预富集与吸附的效果不是很理想,因此提高吸附容量是目前的一大任务。此外,上述已研发的吸附剂有的降解机理不明确,易造成二次污染。综上,易回收、环境友好的农药吸附剂是目前的主要研究方向。其次,目前样品的预富集与吸附和检测方法的发展是相对独立的工作,检测农药残留的方法一般都需要价格昂贵的仪器,所以开发能够减少工作量、降低成本的联用检测机制是未来极具前景的研究方向。最后,基于环糊精的智能吸附剂的研发也是降低检测残留微量农药的成本、减少残留微量农药危险的一个高效快捷的方法,如研发既能高效吸附又可以根据残留农药浓度响应的用肉眼可观察到颜色变化的智能材料。
